# 不同酶处理的啤酒酵母蛋白酶解液功能肽组学分析

**DOI:** 10.3724/SP.J.1123.2023.08029

**Published:** 2023-11-08

**Authors:** Yutong YAN, Chunyu GAO, Xiaomei ZHANG, Zizhe AN, Yunzhen MA, Linlin HAN, Hongwei ZHANG, Xue ZHAO

**Affiliations:** 1.中国海洋大学食品科学与工程学院, 山东 青岛 266003; 1. College of Food Science and Engineering, Ocean University of China, Qingdao 266003, China; 2.青岛海关技术中心, 山东 青岛 266109; 2. Technology Center of Qingdao Customs District, Qingdao 266109, China; 3.山东大学国家糖工程技术研究中心, 山东 青岛 266237; 3. National Glycoengineering Research Center, Shandong University, Qingdao 266237, China; 4.青岛农业大学巴瑟斯未来农业科技学院, 山东 青岛 266109; 4. Bathurst Future Agri-Tech Institute, Qingdao Agricultural University, Qingdao 266109, China

**Keywords:** 超高效液相色谱-高分辨质谱, 肽指纹谱图, 活性肽, 啤酒酵母, ultra-high performance liquid chromatography-high resolution mass spectrometry (UHPLC-HRMS), peptide fingerprint, active peptide, *Saccharomyces pastorianus*

## Abstract

采用超高效液相色谱-高分辨质谱(UHPLC-HRMS)联用技术,结合PEAKS Online 1.7肽组学分析软件和Peptide Ranker、BIOPEP数据库,分析比较了啤酒酵母的中性蛋白酶和木瓜蛋白酶酶解液的肽指纹谱图和活性肽差异,旨在探究不同酶处理的啤酒酵母蛋白酶解液的肽组学差异。PEAKS Online采用de novo测序,相较于传统数据库,更全面地分析得到五肽及其以下寡肽,使样品中的肽段数据更加完整。实验在中性蛋白酶和木瓜蛋白酶的最佳酶解工艺下制备得到啤酒酵母蛋白酶解液,然后进行UHPLC-HRMS分离鉴定及数据检索和分析。研究结果表明,中性蛋白酶、木瓜蛋白酶的酶解产物中分别鉴定出7221和7062条多肽,其中共有肽为980条,而特有肽分别为6241条和6082条,说明两种不同蛋白酶降解产物的肽指纹谱图有很大的差异。使用Peptide Ranker预测出中性蛋白酶和木瓜蛋白酶的酶解产物中有3013和3095条肽为潜在活性肽,其占比分别为41.73%和43.83%。搜索BIOPEP数据库发现,中性蛋白酶和木瓜蛋白酶的酶解产物中有295条和357条活性肽,其中主要是血管紧张素转化酶(ACE)抑制肽、二肽基肽酶Ⅳ抑制肽和抗氧化肽。比较发现,木瓜蛋白酶产物中活性肽数量高于中性蛋白酶产物,但是活性肽的相对含量低于中性蛋白酶产物,本研究更好地揭示了不同蛋白酶对啤酒酵母蛋白肽产品组成的影响。上述结果为啤酒酵母蛋白肽的功能产品开发和啤酒酵母资源的高值化利用提供了参考。

## Abstract (en)

The aim of this study is to explore differences in the peptidomics of *Saccharomyces pastorianus* protein hydrolysates treated with different enzymes. Briefly, differences in the peptide fingerprints and active peptides of neutral protease/papain-hydrolyzed *S. pastorianus* were analyzed using ultra-high performance liquid chromatography-high resolution mass spectrometry (UHPLC-HRMS) combined with PEAKS Online 1.7 analysis software, Peptide Ranker, and the BIOPEP database. Compared to traditional databases, the PEAKS Online uses de novo sequencing for analysis to obtain oligopeptides smaller than pentapeptides. It provides more comprehensive data of the peptide sample. In this study, enzymatic hydrolysates of *S. pastorianus* protein were prepared under the optimum conditions of neutral protease and papain respectively. In total, 7221 and 7062 polypeptides were identified in the hydrolysates of neutral protease and papain, respectively; among these polypeptides, 980 were common to the two enzymes. The 6241 and 6082 unique peptides found in the hydrolysates of neutral protease and papain, respectively, indicated that the peptide fingerprints of the two hydrolysates are quite different. Peptide Ranker predicted that 3013 (41.73%) and 3095 (43.83%) peptides were potentially bioactive in the hydrolysates of neutral protease and papain, respectively. According to the BIOPEP database, neutral protease and papain contained 295 and 357 active peptides, respectively; these peptides were mainly composed of angiotensin converting enzyme (ACE) inhibitors and dipeptidyl peptidase IV inhibitors and antioxidant peptides. The number of active peptides in the hydrolysate of papain was higher than that in the hydrolysate of neutral protease, but the total ion intensity of active peptides in the former was lower than that in the latter. This study revealed the influence of protease type on the composition of enzymatic hydrolysates from *S. pastorianus* protein. The above results provide a reference for the development of functional products of *S. pastorianus* protein peptides and the high-value utilization of yeast resources.

啤酒酵母(*Saccharomyces pastorianus*)是一种由酿酒酵母(*S. cerevisiae*)和真贝酵母(*S. eubayanus*)杂交获得的重要工业生产菌株^[1]^, 2022年我国啤酒生产的副产品酵母粉的总产量约5.35万吨。啤酒酵母可用于生产蛋白质、超氧化物歧化酶(superoxide dismutase, SOD)和酵母抽提物等活性物质,已广泛地应用于饲料、健康食品和调味料中^[2⇓⇓-5]^。啤酒酵母蛋白质含量丰富,是一种便宜、丰富的蛋白质资源^[6]^。目前研究报道表明,酵母蛋白酶解物中发现了抗氧化肽、血管紧张素转化酶(ACE)抑制肽、抗糖尿病肽、抗菌肽、免疫调节肽等,且广泛地应用于健康食品和化妆品中^[7,8]^。

目前,生产酵母蛋白肽主要使用酶解法和发酵法降解酵母蛋白,酶解法主要采用中性蛋白酶、木瓜蛋白酶和胰蛋白酶等高效蛋白酶酶解制备啤酒酵母蛋白肽,酶解工艺以水解度、多肽的相对分子质量、多肽含量等指标来优化^[9⇓-11]^。比较发现,虽然复合酶解可以获得较高的水解度和肽得率,但是深度酶解会导致很多活性肽被水解,不是制备活性肽的最佳方式^[7]^。蛋白酶解产物中多肽种类和结构复杂,酶的种类和酶解条件对蛋白肽的肽指纹谱图和活性肽的影响还缺少系统的研究。对于啤酒酵母而言,已有研究对其特有蛋白质、独特功能以及不同条件下的蛋白质变化等进行了蛋白质组学分析,而对于肽序列及其活性方面的肽组学研究较少。因此酶解工艺与产物组成之间的关系仍然需要进一步系统的研究,对揭示蛋白肽产品的构效关系具有重要作用。

肽组学是蛋白质组学的一个分支,是对生物样品中的肽进行全面定性和定量描述的技术。基于质谱平台的肽组学分析技术有着一定的分析优势:可以获得反映生物样品特质的足够的分子信息且可以进行数据采集后回溯分析;质谱方法具有检测准确性高、在线分离能力强、灵敏度高^[12⇓⇓-15]^以及多目标和高通量分析的优势;多肽的序列结构对于某些加工过程(如加热、剪切等)具有更好的分析稳定性。因此,基于质谱平台的肽组学分析技术已经广泛地应用于蛋白质酶解产物中多肽指纹谱图、鲜味肽和活性肽的分析鉴定^[15⇓-17]^。但是质谱分析的难点在于需要计算和鉴定生物样品中几千或几万个长短肽的序列和含量,工作量非常巨大。

多肽的鉴定最常见的方法是质谱法,其通过对多肽离子质荷比(*m/z*)的分析,测定其相对分子质量和分子结构,依赖现有可用的数据库匹配实验中的质谱数据,实现对多肽的定性分析。并且通过研发一系列联用设备、开发多项新化学技术和优化仪器参数等方法,形成更稳定的“y”或“b”离子来更好地实现质谱数据的匹配。但该法的一个显著缺点是不能用于鉴定来自未知基因组的蛋白质。而基于人工智能算法的PEAKS Online云计算平台,解决了批量质谱数据集高通量解析的难题^[18,19]^。PEAKS Online是一种基于bottom up技术路线的蛋白质组学质谱数据分析软件,采用蛋白质从头测序de novo算法,不依赖于蛋白质数据库,计算所有可能的氨基酸组合中的最佳可能序列,用于在不使用数据库的情况下提取氨基酸序列信息,进行数据转化,对蛋白质进行鉴定及定量分析。研究发现,与目前国际上其他算法平台相比较,PEAKS Online平台可以多解析出5%~30%的肽段序列,把对质谱数据依赖型采集(data dependent acquisition, DDA)和数据非依赖型采集(data independent acquisition, DIA)数据的解析和挖掘能力提升到了一个全新的高度。

本研究采用超高效液相色谱-高分辨质谱(ultra-high performance liquid chromatography-high resolution mass spectrometry, UHPLC-HRMS),结合PEAKS Online云计算平台和BIOPEP、Peptide Ranker在线数据库,分析比较了酵母蛋白的中性蛋白酶和木瓜蛋白酶酶解液的肽指纹谱图和活性肽的差异,揭示了不同蛋白酶对酵母蛋白肽产品组成的影响。

## 1 实验部分

### 1.1 仪器、试剂与材料

Ultimate 3000 UHPLC system色谱仪和Q-Exactive四极杆-轨道阱高分辨质谱仪(美国Thermo公司); ZipTip C18微量层析柱(ZTC18S008)、超滤管(UFC501008)、Milli-Q超纯水制备系统(美国Millipore公司); SIGMA 1-14低温冷冻离心机(德国Sigma公司); PEAKS Online软件(版本1.7,加拿大Bioinformatics Solutions Inc公司);Peptide Ranker在线数据库(distilldeep.ucd.ie/PeptideRanker),用于预测多肽的生物活性;数据库BIOPEP(https://biochemia.uwm.edu.pl/biopep/start_biopep.php),用于预测多肽的生物功能活性。

中性蛋白酶(比活力5×10^4^ U/g)、木瓜蛋白酶(比活力80×10^4^ U/g)均购自北京索莱宝科技有限公司;质谱级甲酸购自美国Sigma-Aldrich公司;质谱级乙腈、质谱级甲醇均购自德国Merck公司。

啤酒酵母粉由青岛啤酒有限公司提供。

### 1.2 样品前处理

啤酒酵母粉以料液比1∶4(g∶mL)加入蒸馏水清洗酵母,3000 r/min离心10 min,收集酵母沉淀,重复洗涤5次。酵母沉淀以料液比1∶4(g∶mL)加入蒸馏水混匀,制成酵母悬浮液。用高压均质机在90 MPa下破壁处理10 min,破壁后的酵母悬浮液分别在中性蛋白酶和木瓜蛋白酶的最佳条件下进行酶解(中性蛋白酶:添加量2000 U/g, pH 7.0,温度50 ℃,时间30 h;木瓜蛋白酶:添加量9600 U/g, pH 6.0,温度50 ℃,时间45 h)。酶解液在100 ℃加热15 min灭酶活后,5000 r/min离心10 min,收集上清液,用10 kDa超滤膜过滤,收集小于10 kDa的滤液为啤酒酵母蛋白酶解液^[20⇓-22]^。

酵母蛋白酶解液用ZipTip C18微量层析柱脱盐,收集乙腈-甲酸(1∶1, v/v)洗脱液,真空干燥。用50%(v/v)乙腈水溶液溶解成1 mg/mL,用0.22 μm超滤膜过滤后,进行UHPLC-HRMS分析。

### 1.3 UHPLC-HRMS条件

色谱条件 色谱柱:C18(150 mm×1.0 mm, 1.7 μm);柱温:40 ℃;流速:0.15 mL/min;进样量:10 μL;进样时间:50 min;流动相:含0.1%甲酸的乙腈(A), 0.1%甲酸水溶液(B);流动相梯度:0~2 min, 5%A; 2~27 min, 5%A~10%A; 27~37 min, 10%A~25%A; 37~39 min, 25%A~80%A; 39~42 min, 80%A; 42~43 min, 80%A~5%A; 43~50 min, 5%A。

质谱条件 电喷雾正离子(ESI^+^)模式;喷雾电压为3.4 kV;在数据依赖型采集模式下,*m/z*扫描范围:200~1500;其他参数设置如下:(1)一级质谱:分辨率70000;扫描阱容量为1×10^6^。(2)二级质谱:分辨率17500;扫描阱容量为1×10^5^;每个采集循环中监测的最强离子数目为20;碰撞能量分别为20、35、45 eV;鞘气(氮气)流速为40 Arb;辅助气体流速为10 Arb;毛细管温度为320 ℃;辅助气体加热温度为300 ℃;信号强度阈值为1.6×10^5^。

### 1.4 多肽质谱数据的分析

使用PEAKS Online软件分析不同酶解产物中所有多肽的一级和二级质谱,根据多肽的二级质谱谱图,采用de novo从头测序算法计算出所有多肽的氨基酸序列。具体搜索设置如下。

模式选择:PEAKS DB;参数设置:前体质量误差(precursor mass error tolerance)15×10^-6^(15 ppm);片段质量误差(fragment mass error tolerance)0.05 Da;酶(enzyme):无;漏切位点(missed cleavage)3;目标数据库(target database)为*Saccharomyces pastorianus*. fasta;置信度(ALC)大于50%;错误发现率(FDR)小于1%。

根据每条肽的提取离子流图计算其离子强度,归一化计算获得其相对离子强度。

### 1.5 多肽生物活性的预测和筛选

将啤酒酵母蛋白酶解物中经PEAKS Online软件解析出的所有多肽序列,使用在线数据库Peptide Ranker预测多肽的生物活性。网站为每个个体肽序列提供了从0到1的生物活性概率,越接近1的肽,其具有生物活性的概率越高,评分大于0.50的多肽被认为具有较高的生物活性^[23]^。使用数据库BIOPEP对多肽的功能活性进行分析^[24]^,该数据库中的肽均为已验证报道的生物活性肽,且随着实验发现不断补充。

## 2 结果与讨论

### 2.1 啤酒酵母蛋白酶解物多肽指纹图谱的分析比较

采用应用广泛且成本低的中性蛋白酶和木瓜蛋白酶对啤酒酵母蛋白进行酶解,因酶解因素之间存在复杂的交互作用,酶解产物因酶解方法及酶解条件的不同存在很大差异,本文目的在于探究不同酶体系下的酶解产物肽组学差异,因此经过单因素优化,获得最佳酶解条件(见1.2节),并以此制备酶解产物。采用UHPLC-HRMS对酶解产物中的多肽组成和结构进行分析,图1为两种酶解产物中多肽的总离子流图。

**图1:**
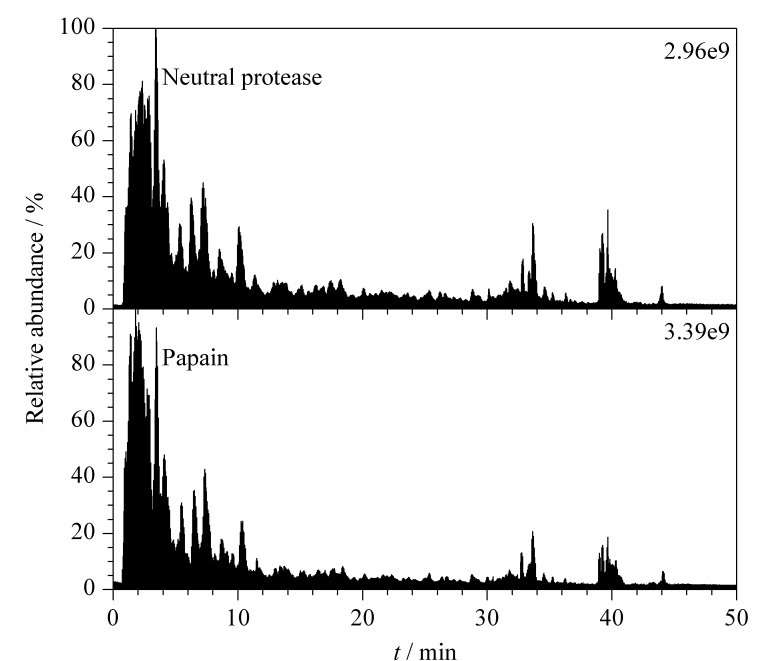
UHPLC-HRMS分析不同啤酒酵母蛋白酶解 产物中多肽的总离子流图

PEAKS Online分析肽序列所采用的方法可以概括为4个步骤:(1)预处理;(2)候选计算;(3)精确评分;(4)全局和位置置信度评分。PEAKS Online基于特征谱峰检测算法来有效地计算最佳肽序列,其片段离子可以很好地解释MS/MS谱中的峰,分析结果可给出具有整个序列的置信度评分的氨基酸序列,以及序列中各个位置氨基酸的评分。与其他分析软件相比,PEAKS基于特征谱峰检测算法,测序快速而准确,可深度挖掘质谱数据;不仅能计算出更多最优的序列和氨基酸,而且还能输出位置置信度得分,从而可靠地确定哪些序列或氨基酸可能性最大^[25]^。

如图2所示,采用PEAKS解析序列为FLSL的四肽,根据特征谱峰所反映的相对分子质量,找到对应的b、y离子以及其他对应离子,y_1_(*m/z*=132.10)、y_2_(*m/z*=219.13)、y_3_(*m/z*=332.21)、b_2_(*m/z*=261.16)、b_3_(*m/z*=348.18)、y_2_-H_2_O、b_2_-NH_3_等,推算出该谱图所对应肽段序列为FLSL,且结果中给出序列FLSL评分为84.7,氨基酸评分分别为89、89、92、68。

**图2:**
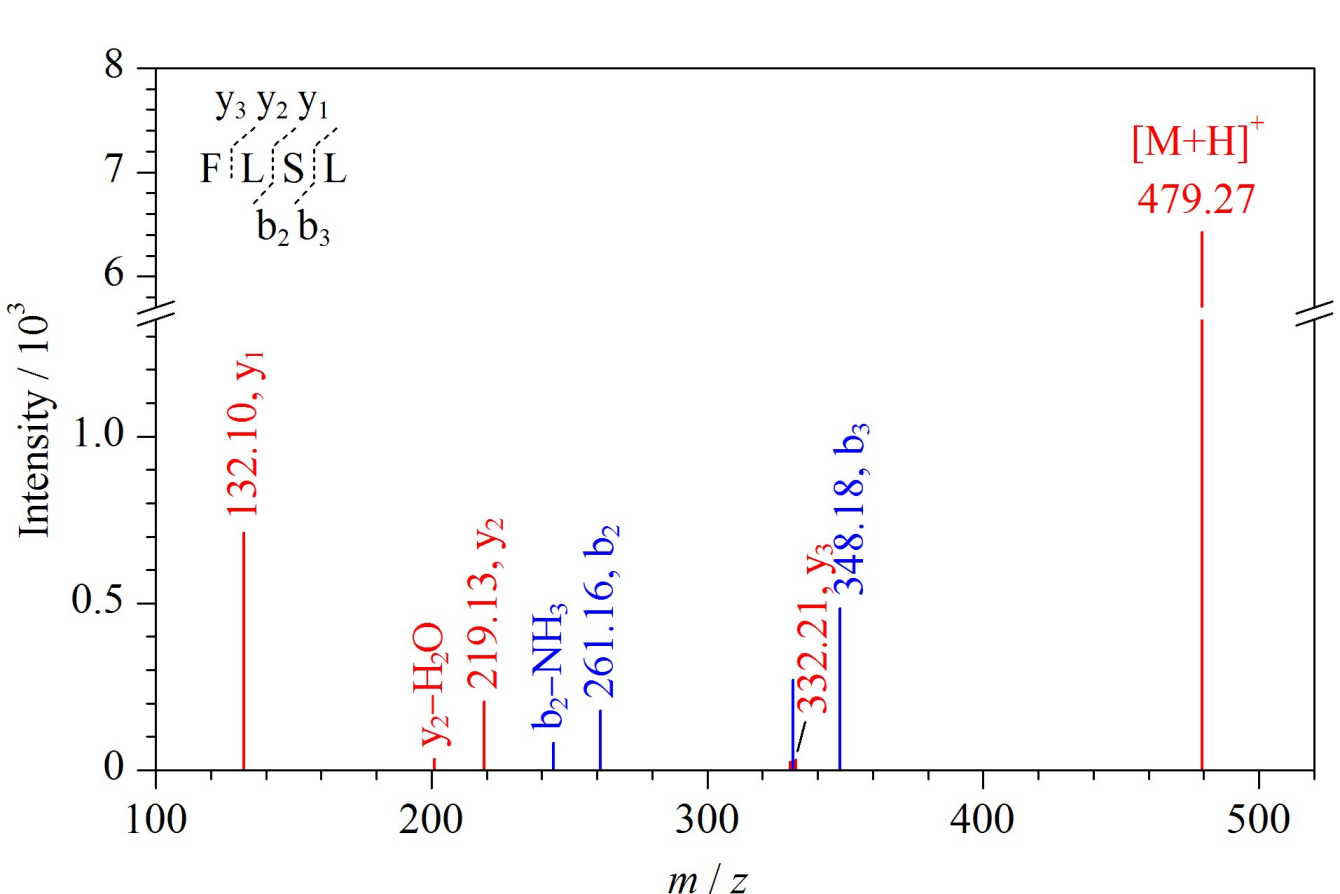
PEAKS Online解析酶解产物中多肽组成示例图

利用多肽的二级质谱和PEAKS Online 1.7软件,从中性蛋白酶和木瓜蛋白酶的酶解产物中分别解析出聚合度2~16的多肽7221条和7062条。表1的多肽组成比较发现,酶解产物中,四肽、五肽和六肽的数量占比较高,其中五肽数量为最多。木瓜蛋白酶酶解产物中三肽和四肽的相对离子强度显著高于中性蛋白酶酶解产物,分别是28.41%和26.90%;而二肽(相对离子强度为22.62%)、六肽及以上多肽的相对离子强度显著低于中性蛋白酶酶解产物,说明木瓜蛋白酶酶解产物中多肽的相对分子质量相对更低。

**表 1:** 不同啤酒酵母蛋白酶解产物中多肽的组成

Peptide composition	Number of common peptides	Specific peptides				
		Neutral protease			Papain	
Number	Relative abundance/%	Number	Relative abundance/%
Amino acid	9	0	1.36		3	5.76
Dipeptide	90	25	30.78		38	22.62
Tripeptide	309	231	18.67		474	28.41
Tetrapeptide	384	1120	21.98		1883	26.90
Pentapeptide	171	1987	13.32		2109	11.62
Hexapeptide	17	1204	6.46		873	2.26
Heptapeptide	6	681	3.39		337	0.84
Octapeptide	2	447	1.82		148	0.72
Nonapeptide	1	206	0.67		79	0.17
Decapeptide	0	133	0.38		43	0.22
More than decapeptide	0	207	1.18		98	0.48
Total	980	6241			6082	

由表1可知,两种酶解产物中共有肽为980条,其中97.35%为2~5肽。而中性蛋白酶酶解产物中特有肽6241条,木瓜蛋白酶的酶解产物中特有肽为6082条,说明两种不同蛋白酶降解产物的肽指纹谱图有很大的差异。

此外,相较于传统蛋白数据库,PEAKS Online采用de novo测序,分析得到更多五肽及其以下寡肽,使其样品中肽段数据更加全面,更好地实现对样品中生物活性肽的预测及筛选。

### 2.2 使用Peptide Ranker对啤酒酵母蛋白酶解物中生物活性肽的预测

Peptide Ranker是一个较为系统的肽数据库,可以评价肽的生物活性概率。Peptide Ranker算法是基于支持向量机(support vector machine, SVM)的机器学习算法。该算法通过训练数据集中的肽序列和对应的生物活性数据,建立一个分类模型。该模型可以根据输入的肽序列,预测其生物活性,并给出一个相应的评分。使用Peptide Ranker数据库对啤酒酵母蛋白酶解产物中所有多肽进行了活性功能预测,活性评分高于0.50则被标记为具有生物活性。

木瓜蛋白酶酶解产物中评分大于0.50的活性肽有3095条,占总肽数目的43.83%,显著高于中性蛋白酶酶解产物中活性肽数目(3013条,占41.73%);其中两种酶解产物的980条共有肽中,评分大于0.50的活性肽有521条,占比53.16%,说明啤酒酵母蛋白酶解产物中有大量的活性肽。表2列举了不同酶解产物中评分较高的10条活性肽的氨基酸序列和质谱解析结果。其中五肽FGFWF占多肽总离子强度的0.1091%,其他活性肽的相对离子强度都低于0.1%,说明单条活性肽在酶解物中的相对离子强度都非常低。

**表 2:** 啤酒酵母蛋白酶解产物中评分最高的10条活性肽的质谱解析结果

Type	No.	Sequence	Score		m/z	z	Relative abundance/%
Neutral protease	1	FFPF	0.9981	557.2792		1	0.0027
	2	FGFWF	0.9981	352.1639		2	0.1091
	3	WWGF	0.9978	595.2611		1	0.0002
	4	WMF	0.9976	483.2065		1	0.0005
	5	WMW	0.9972	522.2168		1	0.0010
	6	WFP	0.9971	449.2188		1	0.0013
	7	WGF	0.9969	409.1870		1	0.0015
	8	PFF	0.9968	410.2078		1	0.0014
	9	FLFF	0.9965	573.3081		1	0.0035
	10	FPMF	0.9965	541.2482		1	0.0002
Papain	1	WFW	0.9988	538.2451		1	0.0050
	2	FMF	0.9977	444.1951		1	0.0022
	3	FPW	0.9971	449.2184		1	0.0056
	4	GFW	0.9970	409.1874		1	0.0040
	5	GFF	0.9970	370.1764		1	0.0213
	6	WPW	0.9968	488.2296		1	0.0015
	7	WLFW	0.9964	651.3292		1	0.0039
	8	FFPGW	0.9963	653.3026		1	0.0001
	9	WDFF	0.9960	614.2609		1	0.0064
	10	FFPM	0.9960	541.2505		1	0.0037

### 2.3 使用BIOPEP对啤酒酵母蛋白酶解物中生物活性肽的预测

使用BIOPEP对酶解产物中鉴定到的所有肽进行活性验证,经过对比分析得到不同酶解物中所含有的生物活性肽,来比较不同酶解产物中活性肽的区别。将PEAKS Online解析得到的啤酒酵母蛋白酶解产物的所有多肽序列输入BIOPEP数据库,搜索已报道的活性肽。附表1为中性蛋白酶和木瓜蛋白酶中筛选出的所有活性肽的氨基酸序列。图3为活性肽的数量和相对离子强度的比较。

**图3:**
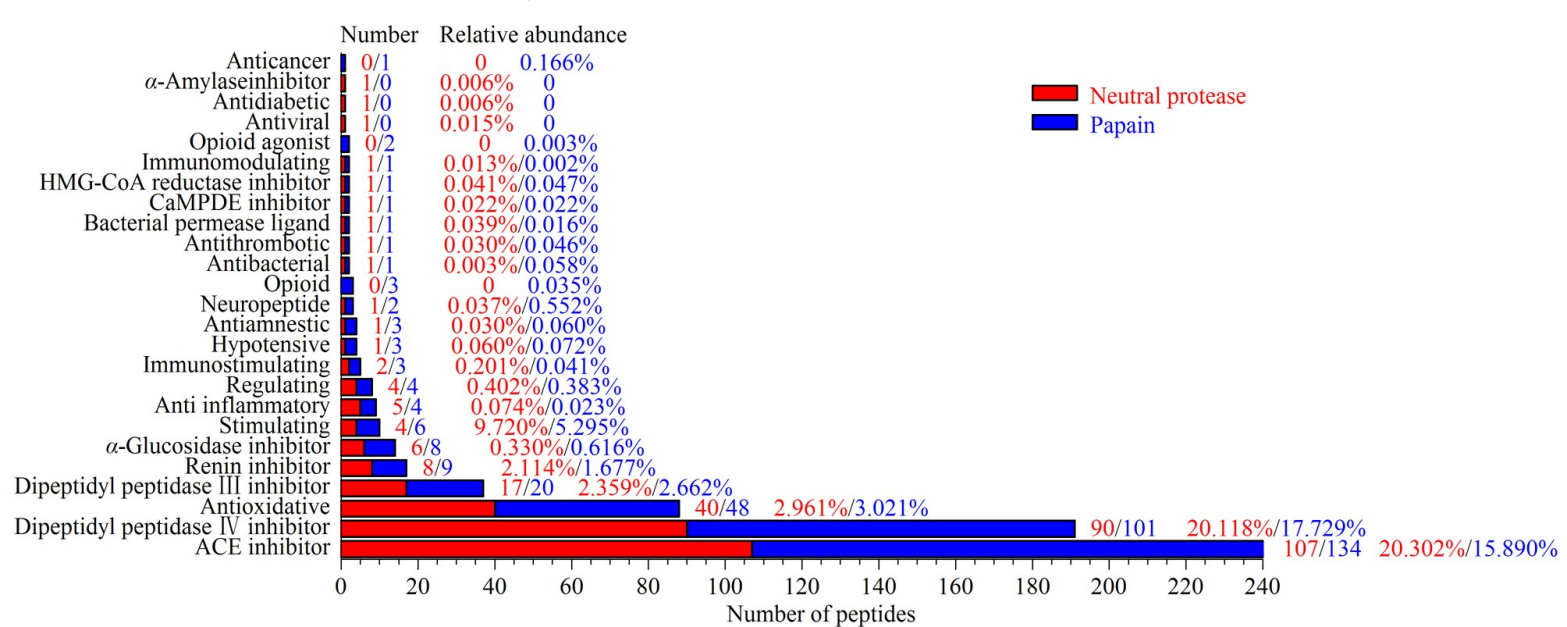
啤酒酵母蛋白酶解产物中活性肽的数量和相对离子强度的比较

中性蛋白酶的酶解产物的7221条多肽中,共发现295条活性肽段(占7221条多肽总离子强度的58.88%),其中有107条为ACE抑制肽,占总肽离子强度的20.30%;90条二肽基肽酶Ⅳ抑制肽,占总肽离子强度的20.12%;40条抗氧化肽,占总肽离子强度的2.96%,17条二肽基肽酶Ⅲ抑制肽,占总肽离子强度的2.36%;8条肾素抑制肽,相对离子强度为2.11%。从木瓜蛋白酶的7062条多肽中,共发现357条活性肽段,占7062条多肽的总离子强度的48.41%,活性肽数量高于中性蛋白酶,但是活性肽的总含量低于中性蛋白酶产物。其中有134条多肽为ACE抑制肽,占总肽离子强度的15.89%。其次为101条二肽基肽酶Ⅳ抑制肽,占总肽离子强度的17.73%, 48条抗氧化肽占总肽离子强度的3.02%, 20条二肽基肽酶Ⅲ抑制肽占总肽离子强度的2.66%, 9条肾素抑制肽占总肽离子强度的1.67%。比较发现,活性肽在木瓜蛋白酶的酶解产物中含量低于中性蛋白酶产物。酶解产物中少数活性肽有区别:中性蛋白酶酶解产物中有少量的抗病毒肽、抗糖尿病肽、*α*-淀粉酶抑制肽,而木瓜蛋白酶酶解产物中有少量的阿片类肽、阿片类激动肽和抗癌肽。

## 3 结论

本文以啤酒酵母粉为原料,利用中性蛋白酶和木瓜蛋白酶酶解制备酵母蛋白肽,基于UHPLC-HRMS分析和PEAKS Online 1.7计算,从中性蛋白酶和木瓜蛋白酶的酶解产物解析出聚合度2~16的多肽有7221条和7062条,其中共有肽980条,中性蛋白酶特有肽6241条,木瓜蛋白酶特有肽6082条,说明两种酵母蛋白酶解产物的肽指纹谱图有很大的差异。使用Peptide Ranker预测两种酶解液中的活性肽,中性蛋白酶和木瓜蛋白酶的酶解产物中分别有3013条和3095条肽为潜在的活性肽。搜索BIOPEP数据库,发现中性蛋白酶和木瓜蛋白酶的酶解产物中分别有295条和357条活性肽段,占总肽离子强度的58.88%和48.41%,其中ACE抑制肽和二肽基肽酶Ⅳ抑制肽为主要活性肽。比较发现,木瓜蛋白酶产物中活性肽数量高于中性蛋白酶,但是活性肽的相对离子强度却低于中性蛋白酶产物。不同蛋白酶生产的酵母蛋白肽产品的肽指纹谱图和活性预测的比较,为筛选高活性酵母蛋白肽产品提供了结构基础,有利于进一步挖掘酵母蛋白肽中的活性肽。
